# Efficient production of 1,2,4-butanetriol from corn cob hydrolysate by metabolically engineered *Escherichia coli*

**DOI:** 10.1186/s12934-024-02317-0

**Published:** 2024-02-12

**Authors:** Ping Li, Mengjiao Wang, Haiyan Di, Qihang Du, Yipeng Zhang, Xiaoxu Tan, Ping Xu, Chao Gao, Tianyi Jiang, Chuanjuan Lü, Cuiqing Ma

**Affiliations:** 1 State Key Laboratory of Microbial Technology, Shandong University, NO.72 Binhai Road, Qingdao, 266237 China; 2 Bloomage Biotechnology Corporation Limited, 678 Tianchen Street, Jinan, 250101 China; 3 Shandong Institute of Metrology, Jinan, 250014 China; 4 State Key Laboratory of Military Stomatology and National Clinical Research Center for Oral Diseases and Shaanxi Key Laboratory of Stomatology, School of Stomatology, The Fourth Military Medical University, Xi’an, 710032 China; 5 State Key Laboratory of Microbial Metabolism, Shanghai Jiao Tong University, Shanghai, 200240 China; 6 School of Municipal and Environmental Engineering, Shandong Jianzhu University, Jinan, 250101 China

**Keywords:** Corn cob hydrolysate, 1,2,4-butanetriol, Xylose, Metabolic engineering, *Escherichia coli*

## Abstract

Corn cob is a major waste mass-produced in corn agriculture. Corn cob hydrolysate containing xylose, arabinose, and glucose is the hydrolysis product of corn cob. Herein, a recombinant *Escherichia coli* strain BT-10 was constructed to transform corn cob hydrolysate into 1,2,4-butanetriol, a platform substance with diversified applications. To eliminate catabolite repression and enhance NADPH supply for alcohol dehydrogenase YqhD catalyzed 1,2,4-butanetriol generation, *ptsG* encoding glucose transporter EIICB^Glc^ and *pgi* encoding phosphoglucose isomerase were deleted. With four heterologous enzymes including xylose dehydrogenase, xylonolactonase, xylonate dehydratase, α-ketoacid decarboxylase and endogenous YqhD, *E. coli* BT-10 can produce 36.63 g/L 1,2,4-butanetriol with a productivity of 1.14 g/[L·h] using xylose as substrate. When corn cob hydrolysate was used as the substrate, 43.4 g/L 1,2,4-butanetriol was generated with a productivity of 1.09 g/[L·h] and a yield of 0.9 mol/mol. With its desirable characteristics, *E. coli* BT-10 is a promising strain for commercial 1,2,4-butanetriol production.

**Supplementary Information:**

The online version contains supplementary material available at 10.1186/s12934-024-02317-0.

## Introduction

Corn is one of the most important sources of starch in the world. Corn cob is inevitably and massively generated as a by-product of the corn agriculture. Nowadays, corn cob is either burnt as fuel or treated as a waste causing environmental pollution [1,2]. Corn cob can be hydrolyzed into corn cob hydrolysate (CCH) with xylose, arabinose and glucose [1,2]. Numerous efforts have been made to obtain efficient routes for resource utilization of CCH [3–5]. Many value-added chemicals including xylitol, [6] xylonate, [7] and ethanol [8] can be produced from CCH by metabolic engineered microorganisms.

1,2,4-Butanetriol (1,2,4-BT) is a platform chemical with versatile applications [9]. The most important application of 1,2,4-BT is to produce 1,2,4-butanetriol trinitrate (BTTN). BTTN has the advantages of low impact sensitivity, high energy level, high thermal stability, no brittleness at low temperature and high reaction safety, and thus is an excellent substitute for nitroglycerin [10]. In addition, 1,2,4-BT can also be used as the precursor for production of plastic monomers or medicines [11,12]. Currently, 1,2,4-BT is commercially produced through malate reduction with NaBH_4_ under high temperature and high pressure [13]. Harsh reaction conditions, serious pollutions, and difficult downstream purification process restricted the application of this chemical method [13]. Therefore, microbial 1,2,4-BT synthesis from renewable sources has attracted considerable attentions [14–16].

**Fig. 1:**
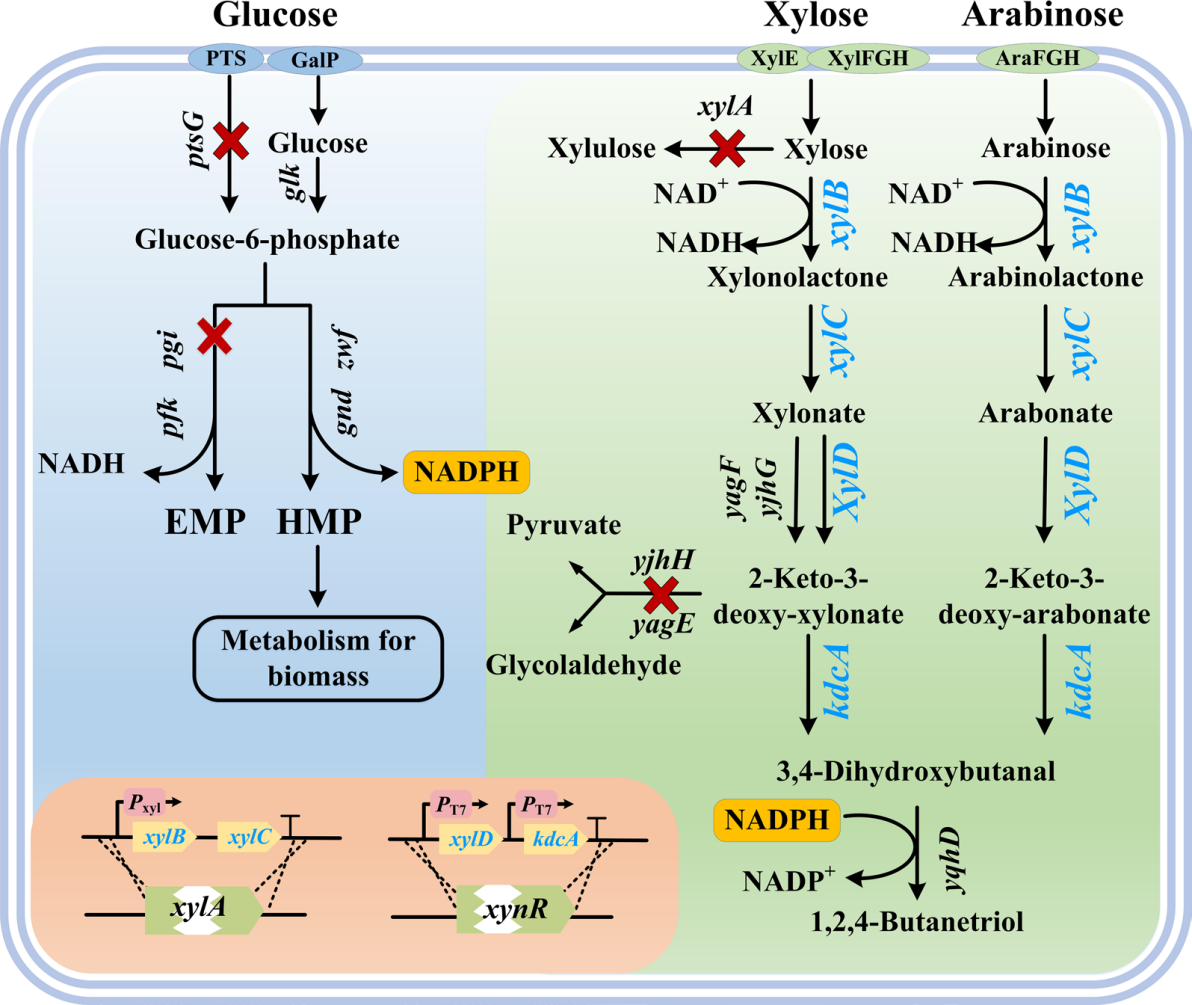
Engineering strategies for 1,2,4-BT production from CCH by recombinant *E. coli*. Red crosses meant that these genes were deleted. Blue typefaces meant that these genes (*xylB*, *xylC* and *xylD* from *Caulobacter crescentus* and *kdcA* from *Lactococcus lactis*) were overexpressed. *xylB*, xylose dehydrogenase coding gene. *xylC*, xylonolactonase coding gene. *xylD*, xylonate dehydratase coding gene. *kdcA*, α-ketoacid decarboxylase coding gene. *xylA*, xylose isomerase coding gene. *yjhG*/*yagF*, xylonate dehydratase coding gene. *yjhH*/*yagE*, 2-keto-3-deoxy-xylonate aldolase coding gene. *yqhD*, NADPH-dependent alcohol dehydrogenase coding gene. *ptsG*, glucose-specific transporter EIICB^Glc^ coding gene. *glK*, glucose kinase coding gene. *pgi*, glucose-6-phosphate isomerase coding gene. *zwf*, glucose-6-phosphate dehydrogenase coding gene. *pfk*, 6-phosphofructokinase coding gene. *gnd*, 6-phosphogluconate dehydrogenase coding gene. EMP, Embden-Meyerhof-Parnas pathway. HMP, Hexose Monophosphate pathway. *xynR*, regulator of xylonate catabolism coding gene. XylE, xylose transporter. AraFGH, arabinose ABC transporter. XylFGH, xylose ABC transporter. GalP, galactose H^+^ symporter. PTS, phosphotransferase system

Researchers have designed different unnatural pathways for 1,2,4-BT biosynthesis from xylose, glucose, arabinose or malate [14–16]. Niu et al. established the first fermentation process for 1,2,4-BT synthesis using two-step fermentation [17]. Xylose or arabinose, the two major pentoses in CCH, are transformed into 1,2,4-BT through a five-enzyme process [17]. The five successive enzymatic reactions can also be introduced into a recombinant strain to realize 1,2,4-BT production [18–22]. Jing et al. blocked the branching pathway of xylose metabolism, fine-tuned the expression of xylose isomerase in *Escherichia coli*, screened an efficient 2-keto-3-deoxyxylonate (2-KDX) decarboxylase KivD, and produced 10.03 g/L 1,2,4-BT from xylose in Luria–Bertani medium [19]. Recently, the group of Sutiono reported the enzymatic synthesis of 1,2,4-BT using xylose as the substrate. 1,2,4-BT production with titers beyond 100 g/L can be acquired under the most suitable conditions [23]. CCH is an ideal substrate for fermentative production of 1,2,4-BT. However, glucose in CCH may induce carbon catabolite repression and inhibit the transport and biotransformation of xylose and arabinose [24]. In addition, glucose catabolism through Embden-Meyerhof-Parnas (EMP) pathway mainly generates NADH but reduction of 3,4-dihydroxybutanal to produce 1,2,4-BT catalyzed by alcohol dehydrogenase YqhD requires NADPH as the cofactor [25]. Herein, *E. coli* W3110 (DE3) was systematically metabolic engineered for generation of 1,2,4-BT from CCH. Briefly, the key genes for production of 1,2,4-BT were overexpressed while the competing pathways related to xylose catabolism were blocked. The *ptsG* encoding the glucose-specific transporter EIICB^Glc^ was knocked out to eliminate carbon catabolite repression and enhance xylose utilization. The *pgi* encoding glucose-6-phosphate isomerase PGI in EMP pathway was deleted to strengthen pentose phosphate pathway and NADPH supply (Fig. 1). Finally, the production of 1,2,4-BT with high concentration, yield and productivity was achieved by the constructed strain *E. coli* BT-10 with CCH as substrate.

## Methods

### Chemicals

Xylose (99%) and lactose were purchased from Shandong Xitang Biotech Co., Ltd. (Jinan, China). 1,2,4-BT was bought from J&K Scientific Ltd (Beijing, China). Xylonate, 2-keto-3-deoxyxylonate (2-KDX), and 3,4-dihydroxybutyrate (3,4-DHBA) were purchased from Sigma-Aldrich (Louis, Missouri, USA). Restriction enzymes were purchased from Thermo Fisher (USA). Polymerase chain reaction (PCR) primers were provided by Beijing Tsingke Biotech Co., Ltd (Qingdao, China). T4 DNA ligase and FastPfu DNA polymerase were purchased from Thermo Fisher (USA) and TransGen Biotech (China), respectively. Whey powder containing 77.0% lactose, 11.2% protein, 1.1% fat, 1.9% moisture, and 8.2% ash was purchased from Kuoquan Biotech (Jinan, China). Detoxified CCH containing 118.5 g/L xylose, 11.8 g/L arabinose, 11.5 g/L glucose, 1.4 g/L formate, 0.34 g/L acetate, 0.83 g/L ethanol and 13.5 mg/L furfural was a kindly gift from Shandong Futase Co., Ltd (Dingtao, China). Other chemicals were analytical grade and commercially available.

### Bacterial strains, plasmids and growth conditions

The bacterial strains and plasmids used in this study are listed in Table 1. *E. coli* strains were generally cultivated in Luria–Bertani (LB) medium (10 g/L tryptone, 5 g/L yeast extract, 10 g/L NaCl) at 180 rpm and 37 °C. The pTKRED and pCP20 were used for the gene knock-out or knock-in of *E. coli* W3110 (DE3). Kanamycin, chloramphenicol and spectinomycin were added at a concentration of 50, 40, and 50 μg/mL when necessary.

**Table 1:** Bacterial strains and plasmids used in this work

Strain or plasmid	Relevant characteristics^a^	Origin
Strain		
*E. coli* DH5α	F^–^ φ80*lacZ*∆M15 ∆(*lacZYA-argF*) U169 *deoR recA1 endA1 hsdR17*(r_K_^–^, m_K_^+^) *phoA supE44* λ^–^ *thi-1 gyrA96 relA1*	Novagen
*E. coli* 0K	*E. coli* W3110 (DE3)	[27]
*E. coli* BT-1	*E. coli* 0K containing plasmid pETP_tac_-*xylBC*	This study
*E. coli* 1K	*E. coli* W3110 (DE3)Δ*xylA*	[27]
*E. coli* BT-2	*E. coli* 1K containing plasmid pETP_tac_-*xylBC*	This study
*E. coli* 3K	*E. coli* 1KΔ*yjhH*Δ*yagE*	[27]
*E. coli* BT-3	*E. coli* 3K containing plasmid pETP_tac_-*xylBC*	This study
*E. coli* 4K	*E. coli* 3KΔ*xynR*	[27]
*E. coli* BT-4	*E. coli* 4K containing plasmid pETP_tac_-*xylBC*	This study
*E. coli* 4KI	*E. coli* W3110 (DE3)Δ*xylA*Δ*yjhH*Δ*yagE*Δ*xynR* with P_T7_-*xylD*-*kdcA* expression cassette knocking in the position of *xynR* in chromosome	[27]
*E. coli* BT-5	*E. coli* 4KI containing plasmid pETP_tac_-*xylBC*	This study
*E. coli* 5KI	*E. coli* 4KIΔ*ptsG*	This study
*E. coli* BT-6	*E. coli* 5KI containing plasmid pETP_tac_-*xylBC*	This study
*E. coli* BT-7	*E. coli* 5KI with P_T7_-*xylBC* knocking in the position of *ptsG* in chromosome	This study
*E. coli* BT-8	*E. coli* 5KI with P_T7_-*xylBC* knocking in the position of *mgsA* in chromosome	This study
*E. coli* BT-9	*E. coli* 5KI with *xylBC* knocking in the position of *xylA* in chromosome	This study
*E. coli* BT-10	*E. coli* BT-9Δ*pgi*	This study
Plasmid		
pETP_tac_-*xylBC*	Expression vector, pETP_tac_ with gene *xylBC*	[7]
pTKRED	Plasmid expressing Red recombinase genes; Spe^r^	Addgene
pCP20	Plasmid expressing Flp recombinase to remove kan cassette; Cm^r^	CGSC^b^
pKD4	Template for kanamycin resistance cassette; Kan^r^	CGSC^b^

^a^*Cm*^*r*^ chloramphenicol resistant, *Km*^*r*^ kanamycin resistant, *Spe*^*r*^ spectinomycin resistant

^b^*CGSC* Coli Genetic Stock Center, Yale university plasmid gene preservation center

### DNA manipulation in *E. coli* W3110 (DE3)

The primers used are listed in Table S1. Vector isolation, restriction enzyme digestion, agarose gel electrophoresis, and other DNA manipulations were carried out using standard protocols as in our previous study [7]. Plasmid was transformed into chemically competent cells by heat shock, and transformants were isolated by plating on antibiotic LB-agar plates. The genes *xylB*, *xylC*, *xylD* and *kdcA* were synthesized by Tongyong Biosystem Co., Ltd. (Chuzhou, Anhui, China) in our previous study [7,27]. The Red recombination technology was applied for knockout of genes *pgi* and *ptsG*, and knock-in of genes *xylBC* from *Caulobacter crescentus* in *E. coli* W3110 (DE3) [26]. The fragment Δ*ptsG* used for knockout of *ptsG* was obtained by directly amplify the fragment with primers Δ*ptsG*-F1/Δ*ptsG*-R1 from *E. coli* K12Δ*ptsG*::*kan* from Coli Genetic Stock Center. The fragment Δ*xylA*::*xylBC* used for knock-in of genes *xylBC* at the position of gene *xylA* was obtained as follows. Primers Δ*xylA*::*xylBC*-F1/Δ*xylA*::*xylBC*-R1, Δ*xylA*::*xylBC*-F2/Δ*xylA*::*xylBC*-R2, Δ*xylA*::*xylBC*-F3/Δ*xylA*::*xylBC*-R3, and Δ*xylA*::*xylBC*-F4/Δ*xylA*::*xylBC*-R4 (Additional file 1: Table S1) were used to clone up homologous arm of *xylA*, the genes of *xylBC*, kanamycin resistance gene, and down homologous arm of *xylA*. These four fragments were recombined to form fragment Δ*xylA*::*xylBC*. The fragments Δ*pgi* for knockout of *pgi*, Δ*ptsG*::P_T7_*-xylBC* and Δ*mgsA*::P_T7_*-xylBC* for knock-in of genes *xylBC* at the position of genes *ptsG* and *mgsA* were obtained through similar process. The fragments for knockout or knock-in of different genes were transformed into *E. coli* cells containing pTKRED plasmid by electrotransformation. Positive transformants were selected by relevant antibiotics and confirmed by PCR and subsequent DNA sequencing. Then, the plasmid pCP20 was transformed to eliminate the kanamycin resistance gene *kan* from chromosome. The two temperature-sensitive plasmids, pTKRED and pCP20, were removed by culture at 42 °C overnight.

### Batch fermentations and fed-batch fermentation

Batch fermentation was carried out in 300 mL shake flasks containing 50 mL of LB medium with 10 g/L xylose and 2 g/L lactose at 180 rpm and 37 °C for 24 h. Xylose at the concentration of 10 g/L was added in the medium for fed-batch fermentation in shake flask when necessary. Fed-batch fermentation was also conducted in 1.0-L bioreactor (Infors AG, Bottmingen, Switzerland) with an operating volume of 0.8 L or 7.5-L (B. Braun Biotech International GmbH, Germany) bioreactor with an operating volume of 5 L, respectively. The seed was inoculated directly into a 35 mL test tube containing 5 mL of the LB medium and then was cultivated at 180 rpm in a rotary shaker at 37 °C overnight. The seed culture was prepared in a 500 mL shake-flask containing 100 mL LB medium, and then seed culture was inoculated (10%, v/v) into the fermentation medium. The LB fermentation medium contained 10 g/L lactose, 10 g/L glucose and 30 g/L xylose. Alternatively, the detoxified CCH (25%, v/v) and whey powder (12.99 g/L) were added into the broth to make the xylose concentration at about 30 g/L and lactose concentration at about 10 g/L. Fermentation was performed at 30 °C with an aeration rate of 1.5 vvm and an agitation of 400 rpm. Samples were withdrawn periodically to determine the cell density, concentrations of glucose, xylose, arabinose, lactose, 1,2,4-BT and by-products. When xylose concentration was lower than 10 g/L, xylose or CCH was added to make xylose concentration to 30 g/L. The pH was maintained at 7.0 by automatic addition of 10 M NaOH.

### Analytical methods

Cell density was determined by monitoring the absorbance at 600 nm using a visible spectrophotometer (V-5100H, METASH, China). The glucose concentration was enzymatically measured using a bioanalyzer (SBA-40D, Shandong Academy of Sciences, China) after appropriate dilution. The concentrations of xylose, arabinose, xylonate, 2-KDX, 3,4-DHBA, 3,4-DHB, 1,2,4-BT and lactose were analyzed using HPLC system (LC-20AT, Shimadzu, Japan) equipped with Aminex HPX-87H column (300 × 7.8 mm, Bio-Rad, USA) linked with an Aminex fast acid analysis column (100 × 7.8 mm, BioRad, USA). The samples withdrawn periodically were centrifuged at 13,000 g for 10 min and the supernatants were filtered using a 0.22 μm Millipore filter (Millipore Corp, Bedford, MA, USA) before HPLC analysis. The 0.1% formic acid was used as the mobile phase at a flow rate of 0.4 mL/min, and the column temperature was 30 °C [27].

## Results and discussion

### Construction of 1,2,4-butanetriol biosynthesis pathway in *E. coli*

**Fig. 2:**
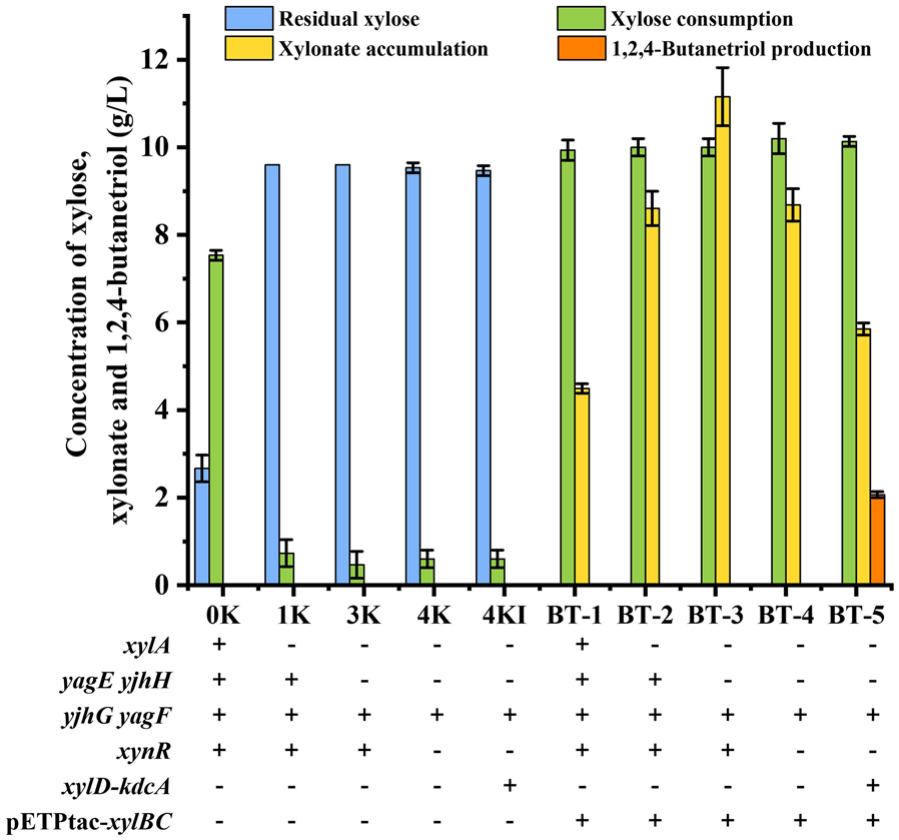
Performance of different recombinant *E. coli* strains in 1,2,4-BT production from xylose. The experiment was carried out in the 300 mL shake flask containing 50 mL LB broth with 10 g/L xylose and 2 g/L lactose at 37 °C and 180 rpm for 24 h. Values are the average ± SD (n = 3 independent experiments)

Biosynthesis of 1,2,4-BT from xylose and arabinose involves five identical enzymic steps including dehydrogenation, hydrolysis, dehydration, decarboxylation and reduction (Fig. 1). In this study, the synthesis pathway for production of 1,2,4-BT from xylose, the most abundant carbohydrate in corn cob, was firstly constructed in *E. coli* W3110 (DE3) (referred to herein as 0K). *E. coli* 1K, 3K, 4K and 4KI were four derivative strains of *E. coli* 0K constructed previously [27]. The *xylA* encoding xylose isomerase was deleted in *E. coli* 1K while the *yjhH* and *yagE* encoding 2-KDX aldolases were further deleted in *E. coli* 3K. To enhance the utilization of xylonate, the *xynR* encoding the regulator of xylonate catabolism was deleted in strain 3K to obtain *E. coli* 4K. A gene cassette for expressing of xylonate dehydratase XylD (YP_002516235.1) of *C. crescentus* and α-ketoacid decarboxylase KdcA (WP_095586306.1) of *Lactococcus lactis* was knocked in *E. coli* 4K at position of *xynR*, resulting in *E. coli* 4KI [27]. Xylose dehydrogenase XylB (YP_002516237.1) and xylonolactonase XylC (YP_002516236.1) from *C. crescentus* can efficiently catalyze xylose into xylonate. The plasmid pETP_tac_-*xylBC* with *xylB* and *xylC *genes from *C. crescentus* was previously constructed and introduced into *E. coli* for efficient production of xylonate from xylose [7]. In this study, the plasmid pETP_tac_-*xylBC* was transformed into *E. coli* 0K, 1K, 3K, 4K and 4KI, resulting in strains *E. coli* BT-1, BT-2, BT-3, BT-4 and BT-5, respectively. Expression of *xylB* and *xylC* in pETP_tac_*-xylBC* is under control of P_tac_ and lactose inducible. These recombinant *E. coli* strains were cultivated in LB medium containing 10 g/L xylose and 2 g/L lactose at 37 ℃ and 180 rpm for 24 h. As shown in Fig. 2, *E. coli* BT-1, BT-2, BT-3, BT-4 and BT-5 with *xylB* and *xylC* exhibited obviously xylose utilization and xylonate production. Endogenous alcohol dehydrogenase YqhD in *E. coli* could reduce 3,4-DHB to 1,2,4-BT [28]. However, only the strain *E. coli* BT-5, in which the genes *xylD* and *kdcA* responsible for 3,4-DHB production from xylonate were knocked in, has the ability for 3,4-DHB production from xylonate. Thus, no accumulation of 1,2,4-BT was observed during culture of *E. coli* BT-1, BT-2, BT-3, and BT-4. 1,2,4-BT at a concentration of 2.06 g/L and a yield of 0.29 mol/mol xylose was accumulated within 24 h by *E. coli* BT-5 (Fig. 2).

### Knockout of *ptsG* to increase 1,2,4-butanetriol production

**Fig. 3:**
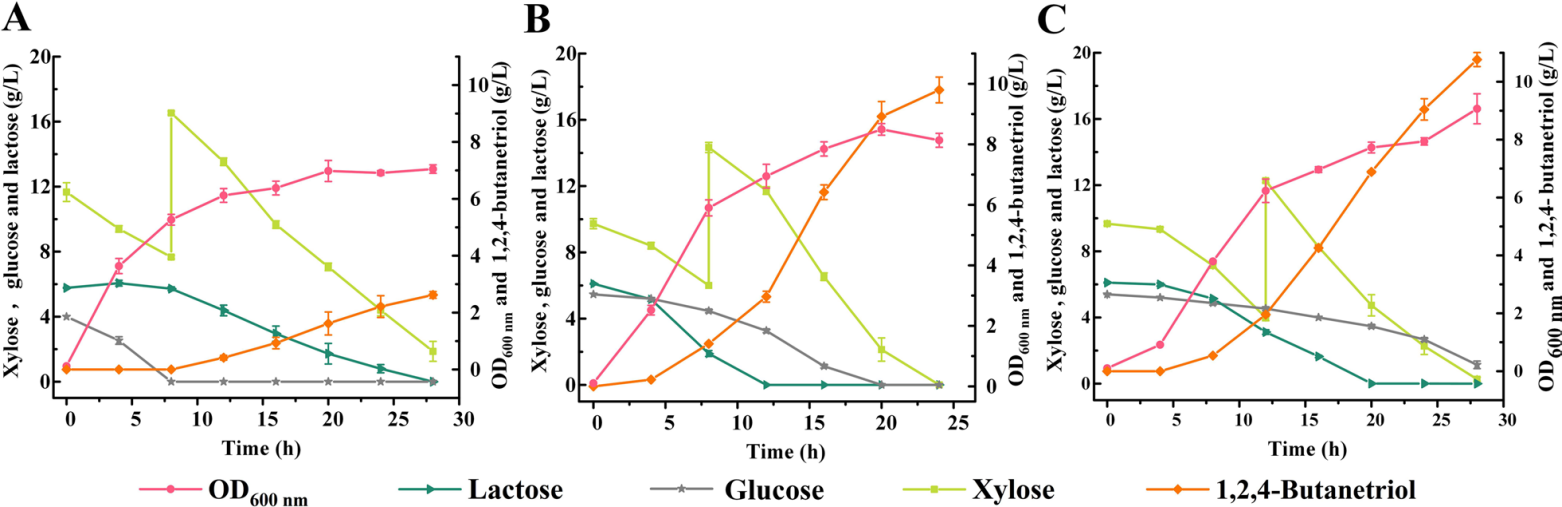
Effect of gene *ptsG* deletion on production of 1,2,4-BT from xylose. **A** Time-course of fed-batch fermentation in the 300 mL shake flask by *E. coli* BT-5 at 180 rpm and 37 °C. **B** Time-course of fed-batch fermentation in the 300 mL shake flask by *E. coli* BT-6 at 180 rpm and 37 °C. **C** Time-course of fed-batch fermentation in 300 mL shake flask by *E. coli* BT-6 at 180 rpm and 30 °C. Xylose at the concentration of 10 g/L was added in the medium when necessary. Values are the average ± SD (n = 3 independent experiments)

Besides xylose and arabinose, glucose which can support *E. coli* growth is also present in CCH. Thus, *E. coli* BT-5 was cultured in LB with 5 g/L glucose and 10 g/L xylose where glucose was utilized to support *E. coli* BT-5 growth and xylose was used for 1,2,4-BT synthesis. When xylose concentration was lower than 5 g/L, 10 g/L xylose was added in the broth. As shown in Fig. 3A, 2.62 g/L 1,2,4-BT was obtained from 18.67 g/L xylose by *E. coli* BT-5 with a yield of 0.20 mol/mol xylose.

*E. coli* preferentially utilizes glucose in medium containing glucose and other utilizable sugars like xylose and lactose [24]. Knocked out the *ptsG* encoding the glucose-specific transporter EIICB^Glc^ can eliminate carbon catabolite repression of glucose. Thus, *E. coli* 5KI was constructed by deleting *ptsG* in *E. coli* 4KI and then the plasmid pETP_tac_-*xylBC* was transformed, resulting in strain *E. coli* BT-6. Glucose can also be transported by galactose symporter after deletion of *ptsG* gene but the glucose utilization rate of the constructed strain *E. coli* BT-6 decreased due to the knocked out of *ptsG* (Fig. 3B). The major byproduct of *E. coli* BT-5 was xylonate (12.30 g/L) (Additional file 1: Figure S1), which can be transformed by lactose induced XylD and KdcA into 3,4-DHB and then reduced to 1,2,4-BT. Expression of *xylD* and *kdcA* is under control of P_T7_ in *E. coli* BT5 and BT-6 and lactose inducible. Besides xylose utilization, the catabolism of lactose in *E. coli* is also repressed by glucose due to carbon catabolite repression [7]. Delayed response to induction often occurs during fermentation using lactose as inducer and glucose as carbon source. Although the consumption of xylose was not improved obviously, the inactivation of *ptsG* could enhance the utilization of lactose by *E. coli* BT-6 (Fig. 3B) and thus may also increase the expression of *xylD* and *kdcA*. As shown in Fig. 3B, 9.80 g/L 1,2,4-BT was obtained by *E. coli* BT-6 with a yield of 0.77 mol/mol xylose and accumulation of xylonate decreased to 4.14 g/L (Additional file 1: Figure S1). The enhanced production of 1,2,4-BT by *E. coli* BT-6 may due to the higher expression of *xylD* and *kdcA* by higher lactose utilization. The α-ketoacid decarboxylase KdcA used in this study was from *L. lactis*, whose optimum growth temperature is 30 ℃. The fermentation temperature for 1,2,4-BT production was tentatively adjusted to 30 ℃ and a slightly higher 1,2,4-BT concentration of 10.76 g/L was obtained with a yield of 0.86 mol/mol xylose from 17.67 g/L xylose (Fig. 3C). Thus, the 1,2,4-BT fermentation was conducted at 30 °C in subsequent experiments.

### Integration of xylonate synthesis genes into *E. coli* genome to increase 1,2,4-butanetriol production

**Fig. 4:**
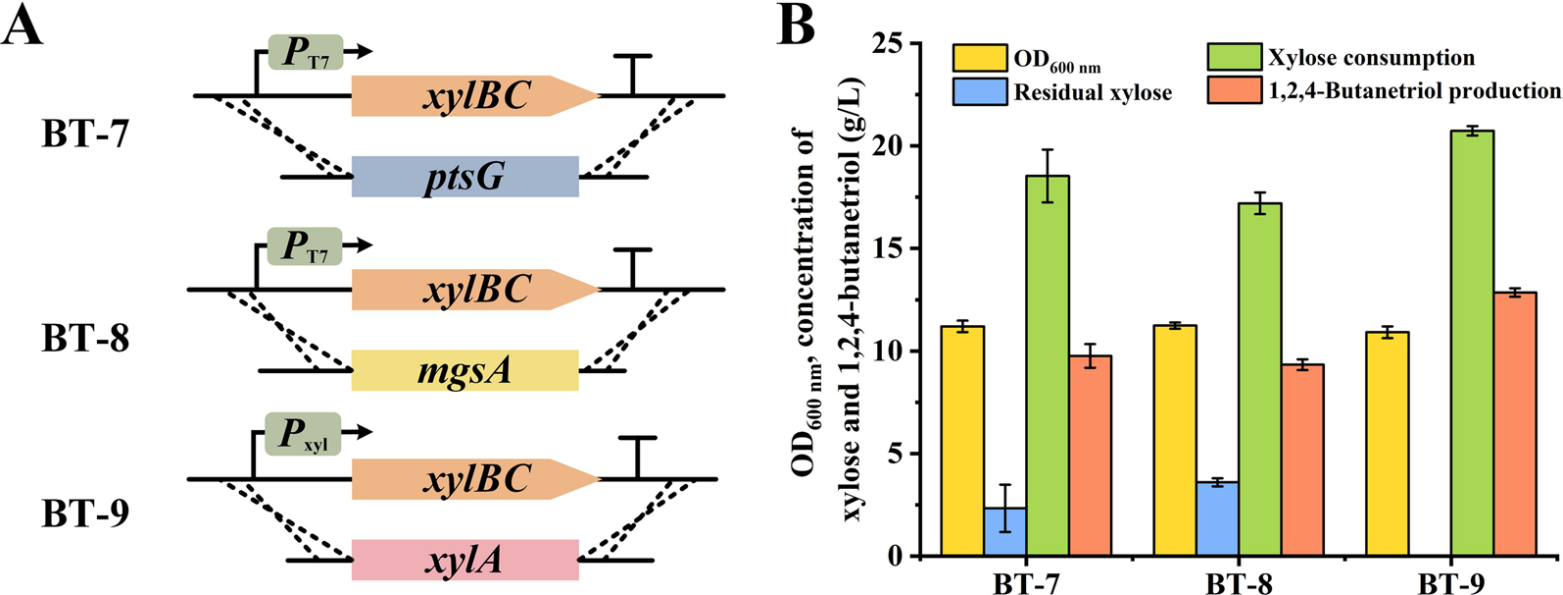
Selection of integration site of *xylBC* to increase 1,2,4-BT production. **A** Scheme of the integration mode of *xylBC* in *E. coli* BT-7, *E. coli* BT-8, and *E. coli* BT-9. **B** Performance of *E. coli* BT-7, *E. coli* BT-8, and *E. coli* BT-9 in transforming xylose into 1,2,4-BT. The fed-batch fermentation was carried out in 300 mL shake flask at 180 rpm and 30 °C. When xylose concentration was lower than 5 g/L, 10 g/L xylose was added. Values are the average ± SD (n = 3 independent experiments)

Expression of exogenous gene based on plasmid may increase the metabolic burden of recombinant *E. coli *[29]. Expression of *xylB* and *xylC* based on plasmid pETP_tac_*-xylBC* may increase the metabolic burden of recombinant *E. coli*. As shown in Fig. 3B, the relief of carbon catabolite repression through deletion of *ptsG* enhanced lactose utilization and increased the production of 1,2,4-BT. It was reported that deletion of *mgsA*, the methylglyoxal synthase coding gene, can also effectively weaken the carbon catabolite repression [30]. Thus, *xylB* and *xylC* under the control of P_T7_ were knocked into the position of *ptsG* and *mgsA* in *E. coli* 5KI genome for simultaneously relieving catabolite repression and expression of key enzymes for 1,2,4-BT production, resulting in *E. coli* BT-7 and *E. coli* BT-8, respectively. The endogenous xylose isomerase coding gene *xylA* is under the control of P_xyl_ and can be induced by xylose in *E. coli *[31]. The *xylB* and *xylC* were also directly knocked into the position of *xylA* and under the control of P_xyl_ in *E. coli* 5KI, resulting in strain *E. coli* BT-9 (Fig. 4A). As shown in Fig. 4B, the strain *E. coli* BT-9 exhibited the best performance in 1,2,4-BT production. 1,2,4-BT at a concentration of 12.85 g/L was obtained from 20.7 g/L xylose with a yield of 0.88 mol/mol xylose in 24 h (Additional file 1: Figure S2).

### Fed-batch fermentation of *E. coli* BT-9 to produce 1,2,4-butanetriol in 1-L bioreactor

**Fig. 5:**
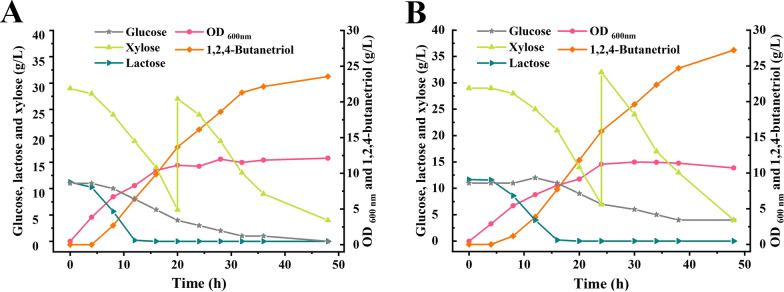
Effect of *pgi* deletion on 1,2,4-BT production from xylose. **A** Time-course of fed-batch fermentation by *E. coli* BT-9 in 1-L bioreactor. **B** Time-course of fed-batch fermentation by *E. coli* BT-10 in 1-L bioreactor. The fed-batch fermentation was conducted in 1-L bioreactor containing 0.8 L LB broth with 30 g/L xylose, 10 g/L glucose and 10 g/L lactose at 400 rpm, 1.5 vvm and 30 °C. Xylose concentration was adjusted to 30 g/L when lower than 10 g/L. The experiments were conducted in triplicate. Two representative time-courses of *E. coli* BT-9 **A** and *E. coli* BT-10 (**B**) are reported herein

Then, fed-batch fermentation was carried out to produce 1,2,4-BT with *E. coli* BT-9. *E. coli* BT-9 was cultured in a 1-L bioreactor with 30 g/L xylose, 10 g/L lactose and 10 g/L glucose. Solid xylose was added under non-sterile condition when xylose concentration was lower than 10 g/L. After 48 h fed-batch fermentation, 1,2,4-BT at a concentration of 23.55 g/L was obtained from 46 g/L xylose with a yield of 0.72 mol/mol xylose. (Fig. 5A).

### Knockout of *pgi* to strengthen NADPH supply and 1,2,4-butanetriol production

**Fig. 6:**
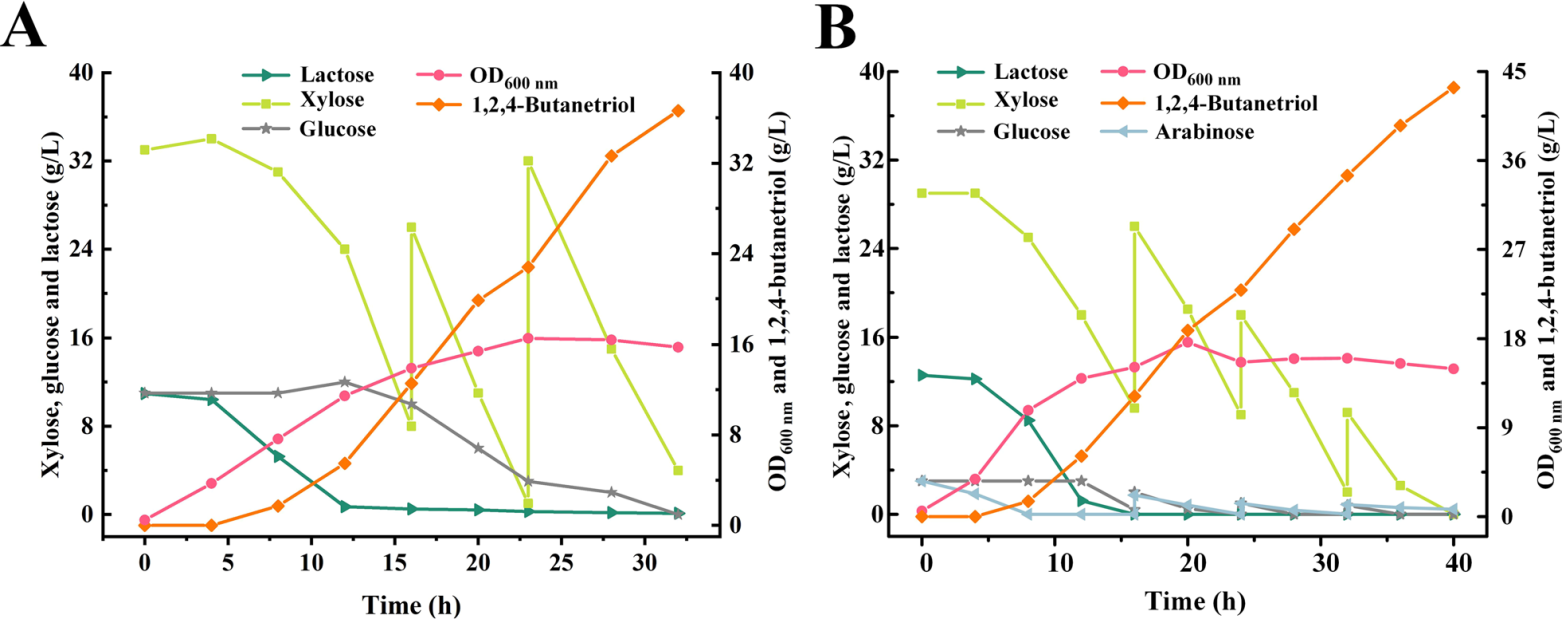
1,2,4-BT production through fed-batch fermentation with *E. coli* BT-10 using xylose or CCH as the substrate. **A** Time-course of fed-batch fermentation by *E. coli* BT-10 in 7.5-L bioreactor with xylose as the substrate. The experiment was conducted in 7.5-L bioreactor with 5 L LB broth containing 30 g/L xylose, 10 g/L glucose and 10 g/L lactose at 400 rpm, 1.5 vvm and 30 °C. Xylose concentration was adjusted to 30 g/L when lower than 10 g/L. The experiment was conducted in triplicate. A representative time-course is reported herein. **B** Time-course of fed-batch fermentation by *E. coli* BT-10 in 7.5-L bioreactor with CCH as the substrate. The experiment was conducted in 7.5-L bioreactor containing 5 L LB broth with CCH (the final concentration of xylose was adjusted to 30 g/L) and whey powder (the final concentration of lactose was adjusted to 10 g/L) at 400 rpm, 1.5 vvm and 30 °C. Xylose concentration was adjusted to 30 g/L when lower than 10 g/L. The experiment was conducted in triplicate. A representative time-course is reported herein

XylB catalyzes xylose into xylonolactone and generate NADH. However, YqhD required for 1,2,4-BT production is NADPH-dependent. Glucose can enter into EMP pathway to produce NADH or enter into pentose phosphate pathway to produce NADPH. Thus, the *pgi* encoding glucose phosphate isomerase was deleted in *E. coli* BT-9 to block the EMP pathway and enhance glucose metabolism via the HMP pathway to provide more NADPH required for 1,2,4-BT synthesis. The obtained *E. coli* strain BT-10 was used for 1,2,4-BT production in 1-L bioreactor. As shown in Fig. 5B, the 1,2,4-BT production of *E. coli* BT-10 increased to 27.2 g/L with a yield of 0.77 mol/mol xylose. Compared with *E. coli* BT-9, the accumulation of 3,4-DHB decreased 14.58% in *E. coli* strain BT-10 (Additional file 1: Figure S3). Then, the fed-batch fermentation of 1,2,4-BT by *E. coli* BT-10 was conducted in a 7.5-L bioreactor. Both the growth of *E. coli* BT-10 and the xylose consumption rate increased in the 7.5-L bioreactor, which may due to the improved dissolved oxygen during the enlargement of fermentation volume. As shown in Fig. 6A, 36.63 g/L of 1,2,4-BT was produced from 79 g/L xylose with a productivity of 1.14 g/[L·h] and a yield of 0.66 mol/mol xylose.

### Fed-batch fermentation of 1,2,4-BT with detoxified CCH as the substrate

Finally, CCH was used as the substrate for fed-batch fermentation of 1,2,4-BT by *E. coli* BT-10 in a 7.5-L bioreactor. To further reduce the cost for 1,2,4-BT production, lactose was replaced by whey powder (lactose concentration was adjusted to 10 g/L) as the inducer of the genes for 1,2,4-BT production. The protein and fat in whey powder can also be utilized by *E. coli* BT-10 for growth. The biomass of *E. coli* BT-10 increased in the fermentation system with whey powder (Fig. 6A and B), which may improve the transform of xylose and arabinose in CCH for 1,2,4-BT production. As shown in Fig. 6B, *E. coli* BT-10 consumed 62 g/L xylose, 6.16 g/L arabinose and 6.4 g/L glucose in CCH within 40 h. 1,2,4-BT at a concentration of 43.4 g/L was obtained with a productivity of 1.09 g/[L·h]. The yield of 1,2,4-BT from xylose and arabinose was 90% of the theoretical value.

Many biotechnological routes have been developed for the fermentative production of 1,2,4-BT. The synthesis of 1,2,4-BT from xylose or arabinose requires relatively few steps and results in low carbon loss, and thus has been intensively investigated in recent years [16–22,28,32–36]. A series of metabolic strategies such as screening enzymes with high activities, blocking the branch pathways, and enhancing the expression of the key enzymes, have been applied to improve 1,2,4-BT production from xylose or arabinose. CCH containing xylose, arabinose and glucose is an ideal substrate for 1,2,4-BT production. In this work, the encoding genes of XylB, XylC, XylD and KdcA were integrated into the genome of *E. coli* W3110 (DE3). Besides xylose, xylonolactone and xylonate, XylB, XylC and XylD from *C. crescentus* are also active on arabinose, arabinolactone and arabinonate, respectively [37]. KdcA from *L. lactis* catalyzes the decarboxylation of both 2-keto-3-deoxy-xylonate and 2-keto-3-deoxy-arabinonate [38]. Although the catabolic genes for arabinose utilization were not deleted in *E. coli* BT-10, these four heterologous enzymes and endogenous alcohol dehydrogenase YqhD may efficiently redirect the metabolic flux of arabinose from central metabolism to 1,2,4-BT production (Fig. 1). To eliminate catabolite repression and enhance the supply of NADPH for YqhD catalyzed 1,2,4-BT production with glucose, the *ptsG* and *pgi* genes were also deleted in *E. coli* W3110 (DE3). The final recombinant strain *E. coli* BT-10 can produce 43.4 g/L 1,2,4-BT from CCH with a productivity of 1.09 g/[L·h]. Compared with other strains constructed for 1,2,4-BT production (Table 2), *E. coli* BT-10 has significant advantages of high 1,2,4-BT concentration, productivity, yield and efficient utilization of cheap substrate CCH.

**Table 2:** Comparison of 1,2,4-butanetriol production by different microorganisms

Strain	Fermentation method	Substrate	Concentration (g/L)	Yield (mol/mol)	Productivity (g/[L·h])	Reference
*E. coli* BL21(DE3)/pWN6.222A	Batch fermentation in 1-L bioreactor	l-Arabinonic acid	2.4	0.35	NF^a^	[17]
*E. coli* DH5α/pWN6.186A	Batch fermentation in 1-L bioreactor	d-Xylonic acid	1.6	0.25	NF^a^	[17]
*E. coli* EWBT304	Batch fermentation in 300 mL shake flasks	Xylose	0.88	0.13	0.02	[18]
*E*. *coli* as3KXW004	Batch fermentation in 50 mL LB medium	Xylose	10.03	0.47	0.21	[19]
*Saccharomyces cerevisiae* BDδK6035	Fed-batch fermentation in 1.0-L bioreactors	Glucose and xylose	6.60	0.57	0.05	[20]
*S. cerevisiae* BDδD-2tkdcA-ΔBOL2-tTYW1	Batch fermentation in 200 mL-baffled erlenmeyer flask	Glucose and xylose	1.70	0.25	0.02	[21]
*E. coli* R1	Fed-batch fermentation in 50 mL fermentation medium	Glucose and xylose	14.4	NF^a^	NF^a^	[28]
*E. coli* BL21-14	Batch fermentation in 500-mL shake flasks	Xylose	5.1	0.18	0.07	[32]
*E. coli* MJ134k-1	Batch fermentation in 50 mL medium of 250 mL shake flasks	Glucose and xylose	3.96	0.24	0.11	[33]
*E. coli* BL21Δ*xylAB*/pE-*mdlCxylBC*&pA-*adhPyjhG*	Fed-batch fermentation in 5 L-scale bioreactor	Glycerol and xylose	3.92	0.28	0.07	[34]
*E. coli* (pE*tac-mdlC-tac-xyl*B)	Batch fermentation in 20 mL LB medium	Xylose	0.90	0.04	0.02	[35]
*E. coli* W031	Batch fermentation in 5-L bioreactor	Glucose and xylose	3.9	0.3	NF^a^	[36]
*E. coli* BT-10	Fed-batch fermentation in 7.5-L bioreactor	Glucose and xylose	36.63	0.66	1.14	This study
*E*. *coli* BT-10	Fed-batch fermentation in 7.5-L bioreactor	Corn cob hydrolysate	43.40	0.90	1.09	This study

^a^*NF* not found

## Conclusion

In summary, a systematically metabolic engineered strain *E. coli* BT-10 was constructed to produce 1,2,4-BT. 1,2,4-BT at a concentration of 36.63 g/L and a productivity 1.14 g/[L·h] was produced by *E. coli* BT-10 with xylose as the substrate. *E. coli* BT-10 can also use glucose in CCH for growth and transform xylose and arabinose into 1,2,4-BT. 1,2,4-BT of 43.4 g/L was produced from CCH with a productivity of 1.09 g/[L·h]. The process presented here is both a good example for efficient resource utilization of CCH and a promising alternative for industrial 1,2,4-BT production.

### Supplementary Information

**Additional file1: Table S1.** The primers used in this study. **Figure S1.** Effect of gene *ptsG* deletion on byproducts generation during 1,2,4-BT production from xylose. **Figure S2.** Selection of integration site of *xylBC* to increase 1,2,4-BT production. **Figure S3.** Effect of *pgi* deletion on byproducts generation during 1,2,4-BT production from xylose.

## Data Availability

All data generated or analyzed during this study are included in this published article.

## Abbreviations

1,2,4-BT: 1,2,4-Butanetriol
CCH: Corn cob hydrolysate
BTTN: 1,2,4-Butanetriol trinitrate
2-KDX: 2-Keto-3-deoxyxylonate
3,4-DHB: 3,4-Dihydroxybutanal
3,4-DHBA: 3,4-Dihydroxybutyrate
EMP: Embden-meyerhof-parnas pathways
HMP: Hexose monophosphate pathway
