# Supplementary material for: Efficient production of 1,2,4-butanetriol from corn cob hydrolysate by metabolically engineered Escherichia coli
Source: Microb Cell Fact. 2024 Feb 12;23:49. doi: 10.1186/s12934-024-02317-0 (PMC10863244; doi:10.1186/s12934-024-02317-0)
Supplement: Supplementary file 1 — Additional file1: Table S1. The primers used in this study. Figure S1. Effect of gene ptsG deletion on byproducts generation during 1,2,4-BT production from xylose. Figure S2. Selection of integration site of xylBC to increase 1,2,4-BT production. Figure S3. Effect of pgi deletion on byproducts generation during 1,2,4-BT production from xylose. [file 12934_2024_2317_MOESM1_ESM.docx]

***Supporting Information***

**Efficient production of 1,2,4-butanetriol from corn cob hydrolysate by metabolically engineered *Escherichia coli***

Ping Li^a, b^, Mengjiao Wang^a^, Haiyan Di^a^, Qihang Du^c^, Yipeng Zhang^d^, Xiaoxiao Tan^a^, Ping Xu^e^, Chao Gao^a^, Tianyi Jiang^f^, Chuanjuan Lü^a,^ *, Cuiqing Ma^a,^ *

^a^ State Key Laboratory of Microbial Technology, Shandong University, Qingdao 266237, China

^b^ Bloomage Biotechnology Corporation Limited, 678 Tianchen Street, Jinan 250101, China

^c^ Shandong Institute of Metrology, Jinan 250014, China

^d^ State Key Laboratory of Military Stomatology and National Clinical Research Center for Oral Diseases and Shaanxi Key Laboratory of Stomatology, School of Stomatology, The Fourth Military Medical University, Xi’an 710032, China

^e^ State Key Laboratory of Microbial Metabolism, Shanghai Jiao Tong University, Shanghai 200240, China

^f^ School of Municipal and Environmental Engineering, Shandong Jianzhu University, Jinan 250101, China

*Corresponding Authors:

Chuanjuan Lü, E-mail: chuanjuanlv@sdu.edu.cn, Address: NO.72 Binhai Road, 266237, Qingdao, P. R. China, Tel.: +86-532-58631559

Cuiqing Ma, E-mail: macq@sdu.edu.cn, Address: NO.72 Binhai Road, 266237, Qingdao, P. R. China, Tel.: +86-532-58631561

**Table S1** The primers used in this study

| **Primer** | **Sequence (5’-3’)** |
| --- | --- |
| Primers for direct amplification of knock-out fragments | |
| Δ*ptsG*-F | GTTTCACATCGACGCTTCCC |
| Δ*ptsG*-R | TGCCTGTCATGCCAGAGTTG |
| Δ*pgi*-F | ATATCTGGCTCTGCACGACC |
| Δ*pgi*-R | CTTTAGTCGTGGCTGAACAG |
| Primers for recombinant PCR amplification of knock-out fragments | |
| Δ*xylA*::*xylBC*-F1 | CGGAACAATATCGACCAGGGCTTTT |
| Δ*xylA*::*xylBC*-R1 | CTGGGATAGATGGCTGAGGACATATTGAACTCCATAATCAGGTAAT |
| Δ*xylA*::*xylBC*-F2 | ATTACCTGATTATGGAGTTCAATATGTCCTCAGCCATCTATCCCAG |
| Δ*xylA*::*xylBC*-R2 | TTCGAAGCAGCTCCAGCCTACACTTAGACAAGGCGGACCTCATGCT |
| Δ*xylA*::*xylBC*-F3 | AGCATGAGGTCCGCCTTGTCTAAGTGTAGGCTGGAGCTGCTTCGAA |
| Δ*xylA*::*xylBC*-R3 | CCAACGGACTGCACAGTTAGCCGATGGGAATTAGCCATGGTCCATA |
| Δ*xylA*::*xylBC*-F4 | TATGGACCATGGCTAATTCCCATCGGCTAACTGTGCAGTCCGTTGG |
| Δ*xylA*::*xylBC*-R4 | CAGGTAACAAAGCACCAGTAAT |
| Δ*ptsG*::P_T7_-*xylBC*-F1 | CTAAAGTACGTCAGCAAGAGTC |
| Δ*ptsG*::P_T7_-*xylBC*-R1 | ATTTCGCGGGATCGAGATCTAATTGAGAGTGCTCCTGAGT |
| Δ*ptsG*::P_T7_-*xylBC*-F2 | ACTCAGGAGCACTCTCAATTAGATCTCGATCCCGCGAAAT |
| Δ*ptsG*::P_T7_-*xylBC*-R2 | AGCAGCTCCAGCCTACACCAAAAAACCCCTCAAGAC |
| Δ*ptsG*::P_T7_-*xylBC*-F3 | GTCTTGAGGGGTTTTTTGGTGTAGGCTGGAGCTGCT |
| Δ*ptsG*::P_T7_-*xylBC*-R3 | TCTCCCCAACGTCTTACGGAATGGGAATTAGCCATGGTCC |
| Δ*ptsG*::P_T7_-*xylBC*-F4 | GGACCATGGCTAATTCCCATTCCGTAAGACGTTGGGGAGA |
| Δ*ptsG*::P_T7_-*xylBC*-R4 | CACGTTATACCAACTGGCGG |
| Δ*mgsA*::P_T7_-*xylBC*-F1 | TTGAACTGGATAATGGACCG |
| Δ*mgsA*::P_T7_-*xylBC*-R1 | TAATTTCGCGGGATCGAGATCTAATGTACATCCGTAGTTAAC |
| Δ*mgsA*::P_T7_-*xylBC*-F2 | GTTAACTACGGATGTACATTAGATCTCGATCCCGCGAAATTA |
| Δ*mgsA*::P_T7_-*xylBC*-R2 | AGCAGCTCCAGCCTACACCAAAAAACCCCTCAAGAC |
| Δ*mgsA*::P_T7_-*xylBC*-F3 | GTCTTGAGGGGTTTTTTGGTGTAGGCTGGAGCTGCT |
| Δ*mgsA*::P_T7_-*xylBC*-R3 | CGTTTGCCACCTGTGCAATAATGGGAATTAGCCATGGT |
| Δ*mgsA*::P_T7_-*xylBC*-F4 | ACCATGGCTAATTCCCATTATTGCACAGGTGGCAAACG |
| Δ*mgsA*::P_T7_-*xylBC*-R4 | TTCAGCGGTGCGCAAATG |


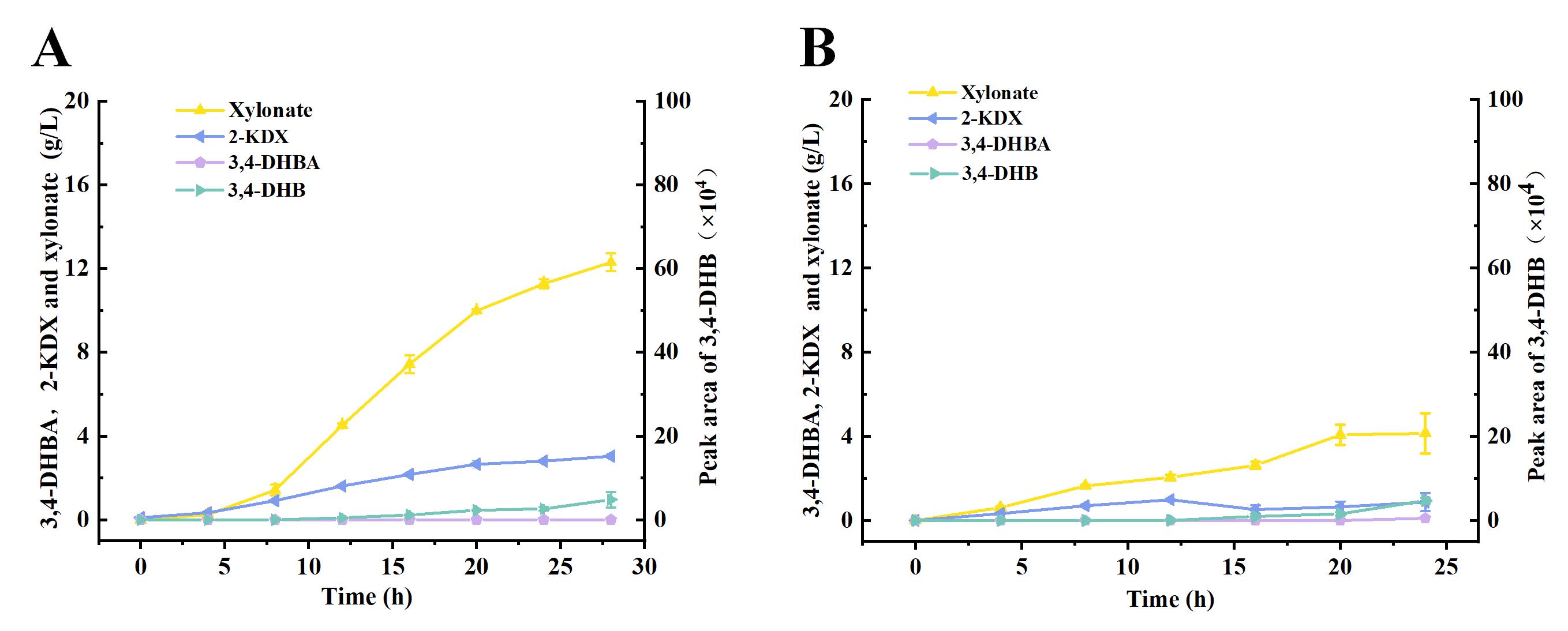


Figure S1 Effect of gene *ptsG* deletion on byproducts generation during 1,2,4-BT production from xylose. (A) Byproducts generation of fed-batch fermentation in the 300 mL shake flask by *E. coli* BT-5. (B) Byproducts generation of fed-batch fermentation in the 300 mL shake flask by *E. coli* BT-6. Xylose at the concentration of 10 g/L was added in the medium when necessary. Values are the average ± SD (n = 3 independent experiments).


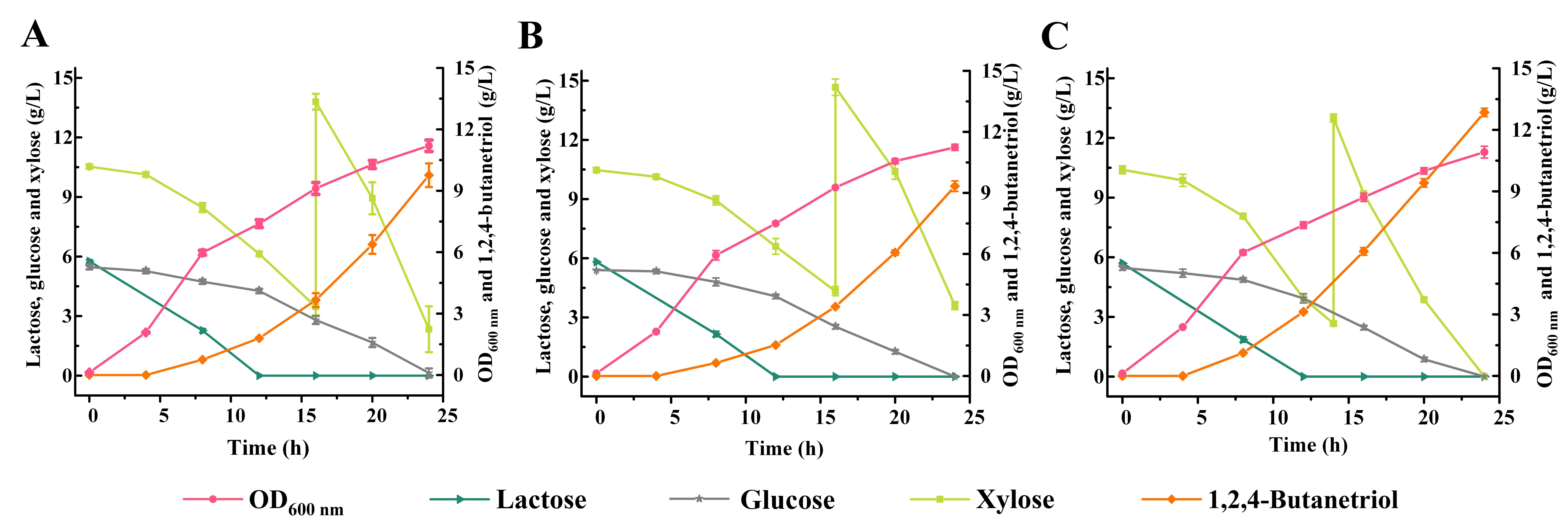


Figure S2 Selection of integration site of *xylBC* to increase 1,2,4-BT production. (A) Time-course of fed-batch fermentation in 300 mL shake flask containing 50 mL LB medium with 10 g/L xylose, 5 g/L glucose and 5 g/L lactose by *E. coli* BT-7 at 180 rpm and 30 °C. (B) Time-course of fed-batch fermentation in 300 mL shake flask containing 50 mL LB medium with 10 g/L xylose, 5 g/L glucose and 5 g/L lactose by *E. coli* BT-8 at 180 rpm and 30 °C. (C) Time-course of fed-batch fermentation in 300 mL shake flask containing 50 mL LB medium with 10 g/L xylose, 5 g/L glucose and 5 g/L lactose by *E. coli* BT-9 at 180 rpm and 30 °C. When the concentration of xylose was lower than 5 g/L, 10 g/L xylose was added in the medium.


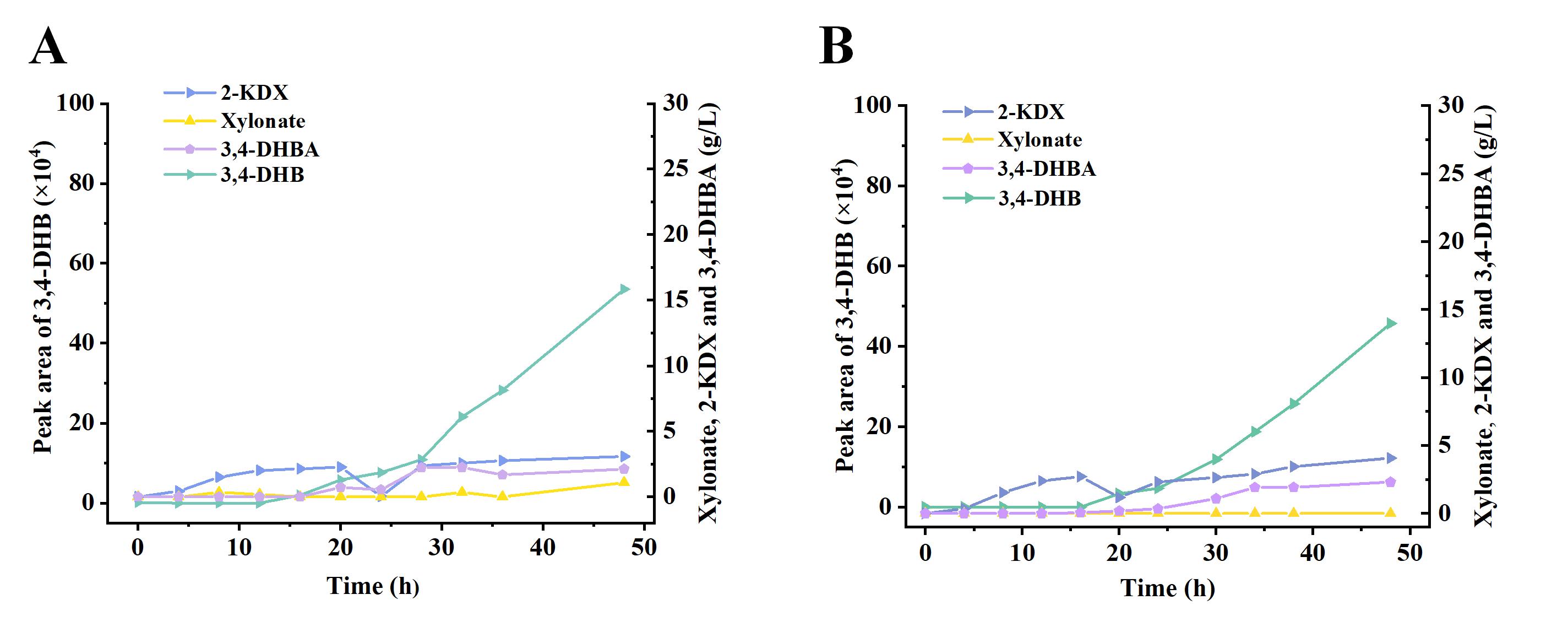


Figure S3 Effect of *pgi* deletion on byproducts generation during 1,2,4-BT production from xylose. (A) Byproducts generation of fed-batch fermentation by *E. coli* BT-9 in 1-L bioreactor. (B) Byproducts generation of fed-batch fermentation by *E. coli* BT-10 in 1-L bioreactor. The fed-batch fermentation was conducted in 1-L bioreactor containing 0.8 L LB broth with 30 g/L xylose, 10 g/L glucose and 10 g/L lactose at 400 rpm, 1.5 vvm and 30 °C. Xylose concentration was adjusted to 30 g/L when lower than 10 g/L. The experiments were conducted in triplicate. Two representative time-courses of *E. coli* BT-9 (A) and *E. coli* BT-10 (B) are reported herein.
